# Modeling ATP-mediated endothelial cell elongation on line patterns

**DOI:** 10.1007/s10237-022-01604-2

**Published:** 2022-07-28

**Authors:** Nicole Roselli, Alessia Castagnino, Giuseppe Pontrelli, Roberto Natalini, Abdul I. Barakat

**Affiliations:** 1 LadHyX, CNRS, Ecole Polytechnique, Institut Polytechnique de Paris, Palaiseau, France; 2 Istituto per le Applicazioni del Calcolo “M. Picone”, CNR, Via dei Taurini 19, Rome, 00185 Italy

**Keywords:** Endothelial cells, Line patterns, ATP release, Stochastic differential equations, Model calibration

## Abstract

Endothelial cell (EC) migration is crucial for a wide range of processes including vascular wound healing, tumor angiogenesis, and the development of viable endovascular implants. We have previously demonstrated that ECs cultured on 15-μm wide adhesive line patterns exhibit three distinct migration phenotypes: (a) “running” cells that are polarized and migrate continuously and persistently on the adhesive lines with possible spontaneous directional changes, (b) “undecided” cells that are highly elongated and exhibit periodic changes in the direction of their polarization while maintaining minimal net migration, and (c) “tumbling-like” cells that migrate persistently for a certain amount of time but then stop and round up for a few hours before spreading again and resuming migration. Importantly, the three migration patterns are associated with distinct profiles of cell length. Because of the impact of adenosine triphosphate (ATP) on cytoskeletal organization and cell polarization, we hypothesize that the observed differences in EC length among the three different migration phenotypes are driven by differences in intracellular ATP levels. In the present work, we develop a mathematical model that incorporates the interactions between cell length, cytoskeletal (F-actin) organization, and intracellular ATP concentration. An optimization procedure is used to obtain the model parameter values that best fit the experimental data on EC lengths. The results indicate that a minimalist model based on differences in intracellular ATP levels is capable of capturing the different cell length profiles observed experimentally.

## Introduction

Endothelial cell (EC) migration, whether individual or collective, is critical for several physiological and pathological processes. During embryogenesis, the coordinated movement of ECs gives rise to a primitive circulatory system that subsequently develops into a functional vascular system. In adult organisms, vascular wound healing is modulated by the movement of ECs toward the injured areas, where they play a central role in repairing the damaged tissues. In addition, pathologies such as tumor development and atherosclerosis involve several facets of EC movement (Folkman 1971, 2002). Thus, investigating the migratory behavior of ECs is fundamental for understanding tissue healing and disease development.

By virtue of their position as the inner cellular lining of blood vessels, ECs are in direct contact with blood and circulating cells. Blood flow-derived mechanical stresses are crucial for determining EC shape and orientation, which in turn impact cellular function (Berk et al. 2001; Hahn and Schwartz 2009). In medium and large arteries, ECs are generally elongated and aligned in the direction of the flow field; however, in the proximity of branches and bifurcations, where atherosclerotic lesions preferentially develop, ECs are typically cuboidal and do not exhibit any preferred orientation (Davies et al. 1986).

In the past several years, cell shape and orientation have been shown to also be modulated by lateral walls that physically constrain cellular spreading (Roca-Cusachs et al. 2008; Versaevel et al. 2012) as well as by biophysical cues exerted on the cells’ basal surfaces via substrate patterns that impose directional bias to the cells’ focal adhesion (FA) sites (Lafaurie-Janvore et al. 2016; Natale et al. 2014; Liliensiek et al. 2010; Ray et al. 2017). The capability of controlling EC morphology and orientation by substrate engineering, combined with an understanding of form-function relationships, provides exciting opportunities for optimizing the design of vascular grafts and endovascular devices to ensure improved hemocompatibility and anti-thrombotic outcomes.

A strategy of substrate patterning for regulating EC shape and function is the implementation of planar patterned surfaces with selectively-defined motifs of adhesive and non-adhesive zones where cells are selectively confined (Azioune et al. 2010; Lafaurie-Janvore et al. 2016). This method allows the creation of a system where cell migration is one-dimensional along the length of the adhesive line pattern, thus providing a tool where migration is more controlled and simplified.

Using time-lapse imaging, we have recently shown that ECs cultured on line patterns where cellular adhesion is limited to 15-μm-wide lines that physically confine the cells exhibit very different migration behavior from cells on control unpatterned surfaces (Gusseva 2017). While ECs on unpatterned surfaces exhibit random motion in the absence of flow, cells on line patterns exhibit three distinct migration phenotypes: (a) “running” ECs (RECs) that are polarized and migrate continuously and persistently on the adhesive lines with possible spontaneous directional changes, (b) “undecided” ECs (UECs) that are highly elongated and exhibit periodic changes in the direction of their polarization while at the same time exhibiting minimal net migration, and (c) “tumbling-like” ECs (TECs) that migrate persistently for a certain amount of time but then stop and round up for a few hours before spreading again and resuming migration. Importantly, each of these three phenotypes is associated with a different average cell length profile. In particular, RECs and UECs exhibit broadly uniform lengths in time, but the latter are considerably more elongated than the former. TECs, on the other hand, have a similar length to that of RECs during their migration phase; however, during the tumbling phase, they take on a round shape and thus have lengths that are considerably smaller than those of RECs.

Why adhesive line patterns promote the occurrence of the three EC phenotypes described above remains unknown, but the physical confinement conferred by the line patterns likely plays a major role. In confined environments, cells dynamically coordinate intracellular machinery to generate forces and remodel their cytoskeleton, leading to cellular elongation (Wyckoff et al. 2006; Wolf and Friedl 2011). All of these processes are energy-demanding (Bursac et al. 2005; Balaban 1990) and hence require adequate levels of intracellular adenosine triphosphate (ATP). There is evidence that cells adjust their ATP production rates to meet their energetic demands (Epstein et al. 2017). ATP can also be released by cells in response to external stimuli. In ECs, for instance, fluid dynamic shear stress has been shown to elicit ATP release (Bodin et al. 1991; John and Barakat 2001). In a number of other cell types, membrane strain and cellular elongation are associated with ATP release (Grygorczyk et al. 2013; Takahara et al. 2014; Takahashi et al. 2017). Thus, for ECs on line patterns, the confinement-induced membrane strain and resulting cellular elongation are likely to promote ATP release.

We hypothesize that the different EC length profiles associated with the different migration phenotypes on line patterns reflect differences in intracellular ATP dynamics. We propose that RECs have sufficiently high intracellular ATP concentrations at all times in order to elongate, polarize, and migrate. In contrast, we posit that UECs have an intermediate level of ATP concentration that is sufficiently high for cell spreading and elongation but not for sustained polarization and migration. Finally, TECs are thought to have low levels of intracellular ATP during the rounding-up (tumbling) phase but manage to “recharge their batteries” so that ATP levels recover sufficiently for the cells to eventually elongate, polarize, and migrate during their running phase. This notion is supported by the observation that when intracellular ATP concentration is low, cells shorten (Wysolmerski and Lagunoff 1988; Atkinson et al. 2004; Poncet et al. 2006; Hinshaw et al. 1991) and stop growing until sufficient ATP is produced (Park et al. 2018).

The aim of the present work is to develop a minimalist mathematical model that describes the coupling between intracellular ATP levels and cellular elongation and to use this model to test the hypothesis described above. We specifically wish to explore if changes in intracellular ATP alone are sufficient to produce the experimentally observed different length profiles associated with the three EC migration phenotypes on line patterns. While we and other groups have previously described models of the effect of shear stress on ATP levels at the EC surface (John and Barakat 2001; Gautam et al. 2006; Comerford et al. 2008; Choi and Barakat 2009; Di Costanzo et al. 2018), no models exist to describe the interplay between intracellular ATP levels and cellular elongation.

## Materials and methods

### Experiments

#### Line patterning

**Fig. 1:**
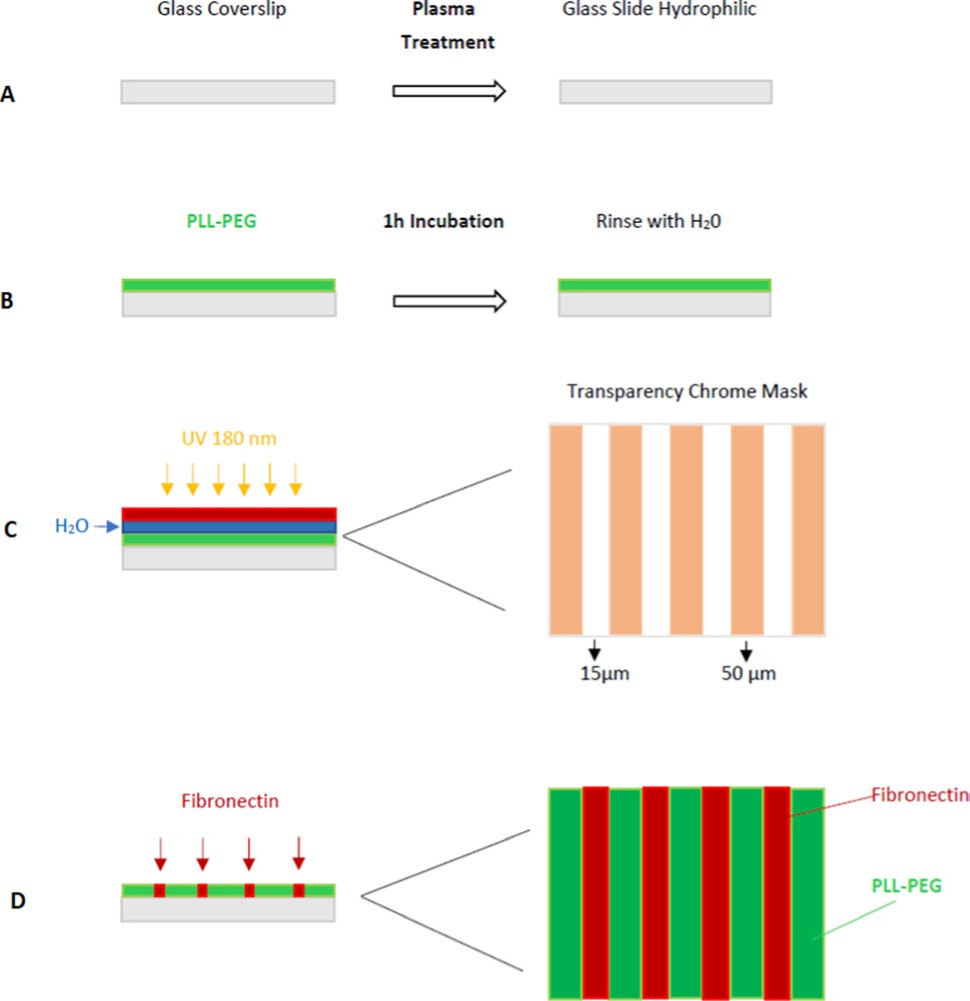
Micropattern fabrication process using the deep UV light method. (1) Glass substrate plasma treatment; (2) PEG coating; (3) UV light treatment; (4) Fibronectin coating. Adapted from Azioune et al. (2010)

Planar micropatterned substrates containing alternating 15-μm-wide adhesive and 50-μm-wide non-adhesive stripes were produced using the deep UV light method (Azioune et al. 2010). Briefly, rectangular and circular glass coverslips were first activated by exposure to air plasma (Harrick Plasma) for 45 s (Fig. 1A). Subsequently, they were incubated for 1 h in 0.1 mg/mL poly-L-lysine-gpoly(ethyleneglycol) (PLL(20)-g[3.5]-PEG(2), SuSoS) in 10 mL HEPES at pH 7.3 for passivation (Fig. 1B). After washing with distilled water, the treated surface was illuminated with deep UV light (UVO-Cleaner, Jelight) through a chromium synthetic quartz photomask (Toppan, TX, USA) for 3 min (Fig. 1C). Unpatterned glass coverslips served as controls. Prior to cell seeding, all patterned and control substrates were incubated for 1 h with 50 $\upmu \text {g}/\text {mL}$ fibronectin solution (Sigma Aldrich Merck KGaA, Darmstadt, Germany) at room temperature (Fig. 1D).

#### Cell culture

Bovine aortic endothelial cells (BAECs, Cell Applications, San Diego, CA, USA; passages 4-8) were cultured in complete Bovine Endothelial Cell Growth Medium (Cell Applications). The cells were incubated at $37^\circ$ in a humidified atmosphere and 5 $\%$ $CO_2$. At confluence, cells were detached with trypsin ($TrypLE^{TM}$ Express Enzyme (1*X*), Gibco, Thermo Fisher Scientific - US) and seeded on patterned and control substrates at a density of $2\times 10^3$ cells/$\text {cm}^2$.

#### Time lapse imaging and image analysis

Image acquisition started 4 h after cell seeding. Time-lapse phase contrast imaging was performed using an inverted microscope (Nikon Eclipse Ti-U, Japan) equipped with a CCD camera (Orca Flash 4.0, Hamamatsu, Shizuoka, Japan), a motorized $x-y$ stage, and “perfect focus” control. The microscope stage was enclosed in an incubator (Okolab, Naples, Italy) allowing control of temperature and *pH*. At least six representative regions per substrate were imaged with a 10x objective using the NIS-Elements Advanced Research software (Nikon). Image acquisition was performed at two different frequencies: every 2 min for 24 h to capture rapid fluctuations in cell length and every 10 min for 12 h to capture global changes in cell length over time.

Cell length measurements over time were performed in FIJI-ImageJ and MATLAB. In each case, 721 frames corresponding to 24 h of migration were used. Colliding cells and cells changing phenotype during the course of a recording were excluded from the analysis. Each of the remaining cells was classified as either a REC, UEC, or TEC, and its length was manually measured at each time point. The analysis was performed on 12 RECs, 13 UECs, and 17 TECs.

### Mathematical modeling

#### Model conceptualization and governing equations

We wish to develop a model that captures the coupling between intracellular ATP levels and EC length dynamics. Conceptually, cellular elongation on line patterns requires ATP and would thus favor ATP production to attain the necessary concentrations. Conversely, the membrane strain that results from cell elongation is expected to elicit ATP release to the extracellular space, thereby reducing intracellular ATP levels. An additional important consideration is that cell elongation requires extensive cytoskeletal remodeling, which requires ATP. For cytoskeletal dynamics, we focus exclusively on filamentous actin (F-actin) because of the implication of actin filaments and stress fibers in the regulation of EC shape.

**Fig. 2:**
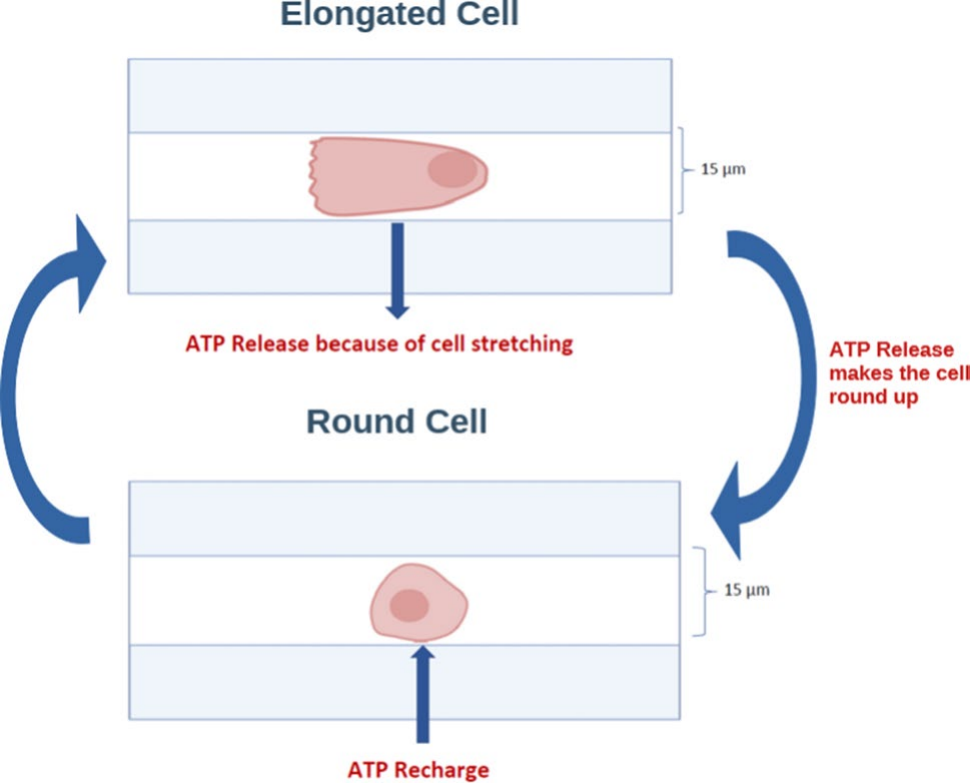
Proposed ATP-driven cyclic tumbling mechanism. Cell elongation elicits ATP release, which decreases intracellular ATP concentration below the level required for cellular elongation, thus leading to cell rounding. Cell rounding reduces ATP release and hence allows ATP production to replenish intracellular ATP to sufficiently high levels to enable cellular elongation, thereby re-initiating the cycle

While cell length remains essentially constant in time for both RECs and UECs and thus the model would need to predict a unique steady state, the situation of TECs is more complex. As depicted schematically in Fig. 2, TECs alternate periodically between elongated (running phase) and round (tumbling phase) states; therefore, the model needs to be able to capture this bistable behavior. To this end, a hysteresis function, dependent on the intracellular ATP level, is used in an analogous manner to what has been done elsewhere (Visintin 1988). As will be detailed below, this hysteresis function posits that an increase in intracellular ATP concentration promotes elongation of the cell, while a decrease in ATP leads to cell shortening. Therefore, cell length will depend on changes in intracellular ATP levels. The model governing equations are described next.

##### F-actin dynamics:

The dynamics of F-actin are assumed to be governed by the following equation and initial condition:

1 $$\begin{aligned} {\left\{ \begin{array}{ll} \dfrac{da}{dt}=k(a_h-a)-aK(c),\\ a(0)=a_{in}, \end{array}\right. } \end{aligned}$$

where *a* and *c* denote the F-actin and intracellular ATP concentrations, respectively. The first term on the right hand side of Eq. (1) represents the rate of F-actin regulation (assembly and disassembly) around the homeostatic F-actin concentration $a_h$; this regulation is assumed to occur with a uniform rate constant *k*. The second term *aK*(*c*) is intended to provide a switch from one F-actin equilibrium state to another, depending on intracellular ATP levels. This formulation is particularly important for the case of TECs where low ATP levels associated with the rounding (tumbling) phase correspond to a reduction in F-actin concentration, whereas high ATP levels associated with the recovery (running) phase correspond to an increase in F-actin concentration. To model *K*(*c*), the equilibria of Eq. (1) for a given value of *c* were first computed as:

2 $$\begin{aligned} a=\dfrac{ka_h}{k+K(c)}. \end{aligned}$$

In Eq. (2), we can then replace the function *K*(*c*) by one of the two constant values $k_1$ and $k_2$, with $k_1>k_2$, to obtain the minimum and maximum possible values of the intracellular F-actin concentration as:

3 $$\begin{aligned} a_{\min }=\dfrac{ka_h}{k+k_1} \end{aligned}$$

and

4 $$\begin{aligned} a_{\max }=\dfrac{ka_h}{k+k_2}. \end{aligned}$$

Based on the notion that F-actin production and elimination are ATP-dependent, the *K*(*c*) function was formulated as follows:

5 $$\begin{aligned} K(c)= {\left\{ \begin{array}{ll} (k_1-k_2)e^{-\frac{c}{M_2(c_h-c)}}+k_2, &{} \text {if }c<c_h,\\ k_2, &{} \text {if }c\ge c_h,\\ \end{array}\right. } \end{aligned}$$

where $c_h$ represents the homeostatic intracellular ATP concentration. With such a formulation, the *K*(*c*) function allows the transition from $a_{\min }$ to $a_{\max }$ as ATP increases. More specifically, when $c\ge c_h$, *K*(*c*) coincides with the constant $k_2$ and so F-actin attains its maximum $a_{\max }$. Conversely, as *c* falls below $c_h$, *K*(*c*) grows rapidly until it reaches the constant value $k_1$, and F-actin attains its minimum $a_{\min }$. The slope of the *K*(*c*) function is modulated by the coefficient $M_2$: the higher the value of $M_2$, the more similar to a Heaviside function *K*(*c*) becomes.

##### Dynamics of cell length:

The formulation for modeling the dynamics of EC length is inspired by the work of Stéphanou et al. (2004) and postulates that cell length is governed by a balance between the effects of cell protrusion due to lamellipodia and filopodia that drive cell extension and intracellular contractility which mediates cellular shortening. Thus, cell length is assumed to be governed by the following equation:

6 $$\begin{aligned} {\left\{ \begin{array}{ll} \dfrac{dL}{dt}=\dfrac{v_p-\sigma (a)L}{\phi }+\beta \xi (t),\\ L(0)=L_{in}, \end{array}\right. } \end{aligned}$$

where $v_p$ is the cell protrusion velocity, which is assumed to be constant, and $\phi$ denotes the friction coefficient between the cell and the substrate. The term $\sigma (a)L$ represents cell contractility as previously formulated by Stéphanou et al. (2004), with $\sigma (a)$ a nonlinear function of the F-actin concentration that has the following form:

7 $$\begin{aligned} \sigma (a)=\psi a^2e^{-a/a_{sat}}. \end{aligned}$$

The function $\sigma$ models the fact that the contractility follows a quasi-quadratic dependence on F-actin concentration until a saturation concentration $2a_{sat}$ after which an effect of compaction of the network occurs and leads to an exponential decrease in contractility. The parameters $\psi$ and $a_{sat}$, represent the contractility constant and the actin saturation concentration, respectively, and they are not directly related to experiments, but characterize the nonlinear contractility function (7) (Stéphanou et al. 2004). Finally, the last term on the right hand side of Eq. (6) represents a stochastic component that describes small-scale fluctuations in cell length that are observed experimentally and that are superimposed on the large-scale changes in cell length due to protrusion and contractility. In this stochastic term, the coefficient $\beta$ represents the standard deviation of cell length which multiplies a Gaussian white noise $\xi (t)$. Note that the intracellular ATP concentration *c* does not appear explicitly in Eq. (6). Rather, its effect on cell length is transmitted through its coupling to the F-actin concentration *a* (see Eq. 1).

Eq. (6) can be written in the differential form

8 $$\begin{aligned} dL(t)=\dfrac{v_p-\sigma (a)L(t)}{\phi }dt+\beta dW(t), \end{aligned}$$

where $dW(t)=\xi (t)dt$ denotes the differential form of the Brownian motion and *L*(*t*) is a one-dimensional Gaussian Ornstein-Uhlenbeck (OU) process (Øksendal 2003).

Equation (8) can be written as:

9 $$\begin{aligned} dL(t)=\alpha (\mu -L(t))dt+\beta dW(t), \end{aligned}$$

where

10 $$\begin{aligned} \alpha =\dfrac{\sigma (a)}{\phi }, \end{aligned}$$

indicates how strongly the system reacts to perturbations and

$$\begin{aligned} \mu =\dfrac{v_p}{\sigma (a)}, \end{aligned}$$

denotes the asymptotic mean of the process. The OU process is mean reverting, meaning that *L*(*t*) reverts to the mean $\mu$ exponentially at rate $\alpha$ with a magnitude proportional to the distance between the current value of *L*(*t*) and $\mu$. Such a property is important because according to how the parameter $\alpha$ is modulated, different solution profiles can be obtained.

The minimum cell length $L_{\min }$ and the maximum cell length $L_{\max }$ are obtained from the experiments, and they are imposed as constraints on the equilibria of Eq. (6). To do so, since the Brownian motion is a zero mean process (${\mathbb {E}}(\xi )=0$), the contribution of the stochastic part can be neglected and the equilibria can be defined through the simple relation:

11 $$\begin{aligned} L=\dfrac{v_p}{\sigma (a)}. \end{aligned}$$

From Eq. (11), it follows that *L* assumes its minimum value $L_{\min }$ when $\sigma (a)$ is at its maximum, i.e., when it is evaluated at $a_{\min }=2a_{sat}$. Imposing this value, it follows that

12 $$\begin{aligned} L_{\min }=\dfrac{v_p}{\sigma (a_{\min })}=\dfrac{v_p}{\psi a^2_{\min }e^{-2}} \end{aligned}$$

and $\psi =\dfrac{v_p}{L_{\min }a^2_{\min }e^{-2}}$. Since $a_{\min }$ maximizes $\sigma (a)$, $\forall a \ne a_{\min }$, it follows that:

13 $$\begin{aligned} L_{\min }<L=\dfrac{v_p}{\sigma (a)}. \end{aligned}$$

Therefore, once the maximum F-actin concentration $a_{\max }$ is identified, the maximum length is readily found through:

$$\begin{aligned} L_{\max }=\dfrac{v_p}{\sigma (a_{\max })}. \end{aligned}$$

##### Dynamics of intracellular ATP concentration:

The intracellular ATP concentration is assumed to be governed by a balance between ATP internal regulation, i.e., net ATP production or elimination, and ATP release into the extracellular space as a result of cellular elongation. Thus,

14 $$\begin{aligned} {\left\{ \begin{array}{ll} \dfrac{dc}{dt}=\lambda (c_h-c)-S_{\max }R(L)H(c),\\ c(0)=c_{in}, \end{array}\right. } \end{aligned}$$

where the first term on the right hand side describes homeostatic ATP regulation with production/elimination rate $\lambda$. The second term on the right hand side represents elongation-induced ATP release where $S_{\max }$ is the ATP release rate and *R*(*L*) and *H*(*c*) are two nonlinear functions whose combination describes the mechanism of ATP release. *R*(*L*) models ATP release due to cell stretching and is assumed to have a sigmoidal behavior as follows:

15 $$ \begin{aligned} R(L)=\bigg (1-e^{{{-M_1}\left(\frac{L}{L_{\min}}{-\xi}\right)}}\bigg )^3, \end{aligned}$$

where $M_1$ is a coefficient that regulates the shape of the function and $\xi$ is a Wiener (stochastic) component. Incorporating a stochastic component into the *R*(*L*) function is needed due to the fact that when solving the overall coupled system of equations, the solution *L* brings stochasticity that needs to be compensated for in order to avoid instabilities in the solution. We note that the function *R*(*L*) used to represent ATP release resembles those commonly used to describe the opening and closing of ion channels (Augustine et al. 1985; Dodge and Rahamimoff 1967; Hubbard et al. 1968), and a sigmoidal behavior has previously been shown to be effective in modeling shear stress-induced ATP release from ECs (John and Barakat 2001).

*H*(*c*) represents a hysteresis function that can be explained by considering the different EC phenotypes that we wish to model. As experimentally observed, RECs and UECs maintain a uniform length in time with only small fluctuations. TECs, on the other hand, exhibit two different equilibria, one associated with the tumbling phase during which the cell is round and remains still and one associated with the recovery phase during which the cell elongates and migrates. In most observations, such episodes repeat in time with a certain periodicity. As already described, we hypothesize that the transition from one equilibrium to the other is driven by changes in intracellular ATP levels. Elevated ATP levels correspond to an elongation of the cell, while low values correspond to cell shortening. In the transition from one state to the other, the system does not trace its steps in reverse; therefore, a hysteresis loop is formed. This is indeed typical of biological systems governed by nonlinear bi-stable processes, where incorporating hysteresis provides an effective strategy for describing the behavior. Examples include switches in protein-DNA interactions (Chatterjee et al. 2008), microscopic cellular signaling pathways with bi-stable molecular cascades (Angeli et al. 2004; Qiao et al. 2007), cell division, differentiation, cancer onset and apoptosis (Eissing et al. 2004; Kim et al. 2007; Wilhelm 2009), protein folding (Andrews et al. 2013), purinergic neuron astrocyte interactions in the brain (Noori 2011), biomechanics of the cornea (Congdon et al. 2006), and lung deformations (Escolar and Escolar 2004). For the current work, hysteresis was formulated in a manner similar to previous work that modeled the growth of concentric rings of bacteria (Hoppensteadt and Jäger 1980; Jäger 1981; Hoppensteadt et al. 1984). These models are based on the notion that forward transitions only occur after a threshold level of the stimulus is reached, while reverse transitions are not initiated below this threshold but rather below a different (lower) threshold. Using this idea, the hysteresis function *H*(*c*) was formulated as follows:

$$\begin{aligned} {H(c)=}{\left\{ \begin{array}{ll} f_1(c),&{} \text {if }\dfrac{dc}{dt}>0 \qquad \qquad \qquad \qquad \qquad \qquad \hfill(16) \\ &{}\\f_2(c),&{} \text {if }\dfrac{dc}{dt}<0,\qquad \qquad \qquad \qquad \qquad \qquad \hfill (17) \,\end{array}\right. } \end{aligned}$$

where the *H*(*c*) function splits in two branches, one (the $f_1(c)$ branch) traversed when the ATP concentration is increasing and the other (the $f_2(c)$ branch) when the ATP concentration is falling. These two branches are associated with the appropriate ATP concentration thresholds $c_L$ which represents the lowest acceptable value for ATP to be in the homeostatic concentration range, and $c_{eq}$ which is an equilibrium ATP concentration that lies between $c_L$ and $c_h$. Specifically, (16) and (17) are defined as follows:

18 $$\begin{aligned} {f_1(c)=}{\left\{ \begin{array}{ll} 0,&{} \text {if }c \le c_{eq},\\ e^{{-\frac{(c_h-c)}{M_3(c-c_{eq})^2}}},&{} \text {if }c_{eq}< c < c_h,\\ 1,&{} \text {if }c \ge c_h.\\ \end{array}\right. } \end{aligned}$$

19 $$\begin{aligned} {f_2(c)=}{\left\{ \begin{array}{ll} 0,&{} \text {if }c \le c_L,\\ e^{{-\frac{(c_{eq}-c)}{M_3(c-c_L)^2}}},&{} \text {if }c_{L}< c < c_{eq},\\ 1,&{} \text {if }c \ge c_{eq}.\\ \end{array}\right. } \end{aligned}$$

**Table 1:** Baseline model parameter values from literature

Symbol	Value	Units	Meaning	Reference
*a*	$0.05-0.2$	mL	F-actin Concentration	Alberts et al. (2002)
$a_{h}$	0.2	mL	F-actin homeostatic value	Alberts et al. (2002)
$c_L$	1	mM	Minimum ATP Concentration	Lodish et al. (2008)
$c_h$	10	mM	Homeostatic ATP Concentration	Lodish et al. (2008)
$v_p$	25	μms^−1^	Protrusion Velocity	Mogilner and Edelstein-Keshet (2002)
$\phi$	3 × 10^−2^	Dimensionless	Friction Coefficient	Angelini et al. (2012)
*k*	347	s^−1^	F-actin Polymerization Rate	Pollard (1986), Mogilner and Edelstein-Keshet (2002)

Some additional comments about the two functions $f_1$ and $f_2$ are warranted:

- *Function* $f_1$: When the cell rounds up, which is a consequence of a drop in intracellular ATP level below the equilibrium threshold $c_{eq}$, it needs to “recharge” its ATP, and since in this particular case there is no ATP release (because there is no membrane stretching), Eq. (14) assumes the simple form: $\dfrac{dc}{dt}=\lambda (c_h-c)$. Thus, the cell is allowed to recover its homeostatic ATP concentration levels. At the same time, with the growth of ATP concentration, cell length also increases. Once the ATP reaches the equilibrium value, the cell starts undergoing membrane stretching, so the function $f_1$ turns from being a zero to a nonzero function, signaling the presence of membrane strain. This means that both terms on the right hand side of Eq. (14) are now nonzero.

- *Function* $f_2$: When the cell is elongated, it releases ATP, and the release is maximum when $f_2=1$ as long as the ATP concentration is higher than $c_{eq}$. Below this value, $f_2$ starts decreasing exponentially until it becomes null when it reaches the minimum admissible ATP threshold $c_L$. From that moment onward, the ATP is allowed to grow again because the sink term has disappeared, and a new hysteresis cycle is established.

When the cell is fully stretched, $L=L_{\max }$ and the ATP is high so that $H=1$ (meaning $f_1=f_2=1$). From the moment the cell reaches the full elongation onward, the intracellular ATP decreases to $c_L$. For this reason, from Eq. 14, we can compute:

20 $$\begin{aligned} \lambda =\dfrac{S_{\max }R(L_{\max })}{c_h-c_L}. \end{aligned}$$

In conclusion, Eqs. (1), (8), and (14) constitute a system of coupled stochastic differential equations (SDEs) whose solution provides the dynamics of F-actin, intracellular ATP concentration, and cell length.

#### Solution methodologies

The governing Eqs. (1), (8), and (14) were solved numerically using a one step IMEX method (Constantinescu and Sandu 2010), which consists of discretizing all terms of the equations implicitly, with the exception of the functions *K*(*c*) and *H*(*c*), which were computed using an explicit approach. In addition, for the OU process and for the ATP release function, which contain stochasticity, the approximation of the Brownian motion as provided by the Euler Maruyama method was applied (Higham 2001). The numerical simulations were performed in MATLAB with time step $\Delta t=10^{-3}$.

#### Parameter values and optimization

The system given by equations (1), (8), and (14) contains several parameters, each of which plays a specific role in determining the dynamics of the three cell states. Some parameter values are obtained either directly or by extrapolation from the literature and are listed in Table 1, while others require the combination of modeling and experiments to be determined. Although most of the unknown values can be found this way, there are still some that remain undetermined. To overcome this problem, the Particle Swarm Optimization (PSO) algorithm (Kennedy and Eberhart 1995) was applied to the system of equations. This algorithm is a metaheuristic global optimization paradigm, which means that it requires few or no assumptions about the problem being optimized, and it can search very large spaces of candidate solutions. The use of classical optimization methods such as the gradient or Newton algorithms encounter difficulties when the problem contains a hysteresis as is the case here. The presence of loops with possible large gradient changes can pose an obstacle to the proper functioning of those methods. Due to its stochastic nature, the swarm optimization technique is able to explore search spaces and to find an optimal solution without the need for assuming global differentiability of the problem.

## Results

### Experimental results

**Fig. 3:**
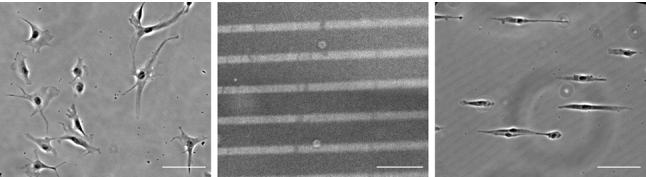
Phase contrast images of BAECs on unpatterned surface (left), pattern coated with fluorescent fibronectin (center) and BAECs on patterned surface (right). Patterning of 15 μm adhesive lines spaced with 50 μm non adhesive lines. Scale bar 100 μm

As shown in Fig. 3 (left), BAECs cultured on a control unpatterned substrate take on many different shapes, are not particularly elongated, and are randomly oriented. An example of the adhesive line patterned surfaces produced using the deep UV patterning protocol described in Methods is shown in Fig. 3 (center), together with an example of BAECs cultured on a patterned surface (right). Cells on the patterned surfaces remain confined to the 15 μm-wide adhesive lines, are often highly elongated, and are uniformly oriented in the direction of the pattern.

**Fig. 4:**
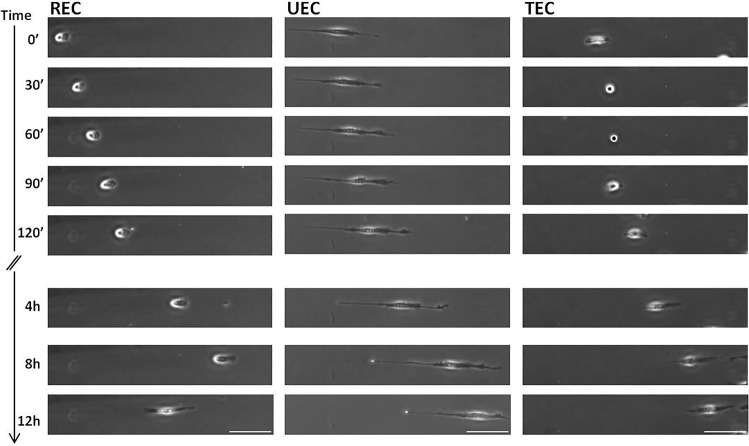
Representative images acquired during cell migration on line patterns. Three categories have been identified: REC (left), UEC (center) and TEC (right). Scale bar 100 μm

Time-lapse imaging allowed visualization of the three EC migration phenotypes described previously, namely RECs, UECs, and TECs. Of particular interest here, these different phenotypes were associated with different cell length profiles. As illustrated in Fig. 4 (left), the lengths of RECs and UECs remained fairly constant throughout the 12 h recording period with fairly small fluctuations and with UECs being considerably more elongated than RECs. TECs exhibited an entirely different behavior with periodic phases of rounding and elongation. During the elongated phase, TECs had lengths that were comparable to those of RECs.

**Fig. 5:**
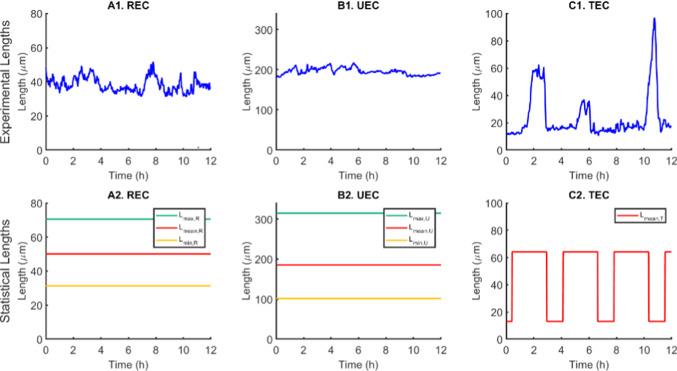
Example of RECs (**A**), UECs (**B**) and TECs (**C**) over a time interval of duration $T=12$ h. Top) Cell lengths as measured from the movies. Measurements are taken every 2 minutes for a total time of 12 hours. Bottom) Statistical lengths obtained from the data processing

**Table 2:** Experimental data for RECs, UECs and TECs

Cell Type	Number of	Maximum	Minimum	Mean	Tumbling	Tumbling	Standard
	Cells	Length	Length	Length	Phase	Interval	Deviation
	$(N_c)$	($L_{\max }$)	($L_{\min }$)	($L_{\text{mean} }$)	($T_P$)	($T_I$)	($\beta$)
RECs	12	70.5 μm	31.4 μm	50.1 μm			1.6 ${\upmu \text{m}} \: \text{s}^{-1/2}$
UECs	13	314.6 μm	101.9 μm	185.5 μm			2.2 ${\upmu } \text{m} \: \text{s}^{-1/2}$
TECs	17	64.1 μm	13.1 μm		1 h $14^\prime$	2 h $33^\prime$	2.1 ${\upmu } \text{m} \: \text{s}^{-1/2}$

For all three phenotypes, the range of length variation was determined, identifying the maximum and minimum lengths (denoted as $L_{\max }$ and $L_{\min }$, respectively). For RECs and UECs, which exhibited largely uniform lengths during the entire recording period, the average length $L_{\text{mean} }$ and its standard deviation were determined. $L_{\text{mean} }$, in combination with the corresponding $L_{\max }$ and $L_{\min }$, was used to design a “statistical” or prototype cell whose average length was given by $L_{\text{mean} }$ and whose length fluctuations were random and bounded by $L_{\min }$ and $L_{\max }$ (Fig. 5 (right) A2 and B2), whereas the standard deviation of the mean length represented the quantity $\beta$ used in the stochastic term in Eq. (8). In the case of TECs, the average duration of the tumbling phase $T_P$ as well as the average time between consecutive tumbling episodes $T_I$ were also computed. We note that a TEC was assumed to be in a tumbling phase when its length fell below 20 μm. This threshold was deemed adequate based on visual inspection of a large number of tumbling episodes. Thus, for TECs, $L_{\min }$, $L_{\max }$, $T_I$, and $T_P$ were combined to generate a prototype length profile as depicted in Fig. 5 (right) C2. The results of the length parameters used to define the prototype cells for all three phenotypes are shown in Table 2.

Figure 5 (right) depicts a representative 12 h recording of a cell from each phenotype along with the corresponding prototype length profile (statistical cell) that the computational model aims to reproduce. While the statistical lengths for RECs and UECs were constant (at 50.1 μm and 185.5 μm, respectively; see Table 2), the behavior of TECs is approximated by means of a square wave with the characteristics given in Table 2. These prototype lengths were employed as classifiers for each phenotype and were used subsequently when applying the optimizer.

**Fig. 6:**
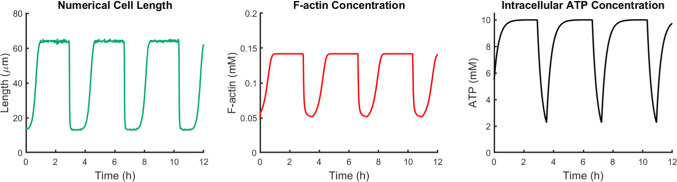
Solution of the system of equations in the case of TECs. Time evolution over $T=12$ h of cell length (left), F-actin concentration (center), intracellular ATP concentration (right)

### Numerical results

#### Parameter estimation from experiments and modeling

We wish to determine the parameter values specific for each of the three cell phenotypes that when inserted in the system of equations, provide numerical solutions that are as close as possible to the experimental measurements of cell length profiles. To this end, we have considered the non-stochastic version of Eqs. (8) and (14).

Equation (1) contains the parameters $k_1$ and $k_2$ that need to be determined. These quantities are, respectively, the rates at which F-actin tends to $a_{\min }$ and to $a_{\max }$ (Eqs. 3 and 4), and they are estimated by combining the modeling with data from literature and experiments (see Appendix). In order to obtain a value for $k_1$, Eq. (3) is used imposing the F-actin range (Alberts et al. 2002) as a constraint. From the resulting relation, it follows that $k_1 = 1.04\times 10^3$ ${\text {s}^{-1}}$. As for $k_2$, Eq. (11) for cell length equilibria is employed. Substituting in this equation the value of $a_{\min }$, which is assumed to be the same for all three migration phenotypes, $L_{\min }$, which has a different value for each phenotype, $L_{\max }$ for TECs and $L_{\text{mean} }$ for RECs and UECs, different $k_2$ values for the different phenotypes are obtained. The full list of computed data is shown in Table 3. It is important to note that while some parameters assume the same values for all phenotypes, others are phenotype-specific, indicating that a first classification of the three types of behavior has been achieved. More details about these computations are provided in the Appendix.

#### Optimization procedure and results

The PSO algorithm was employed with the aim of obtaining the missing parameter values for the system. These are $M_1$, $M_2$, $M_3$, $\lambda$, and $c_{eq}$. The first three are critical for the definition of the slope of the nonlinear functions *R*(*L*), *K*(*c*) and *H*(*c*), while $\lambda$ represents the rate of ATP production and $c_{eq}$ is an equilibrium concentration for ATP.

The model calibration is based on the solution of the following minimization problem:

21 $$\begin{aligned} \min _{\theta \in \Theta }J(\theta ), \end{aligned}$$

where $\theta$ is the vector of the unknown parameters and $J(\theta )$ is defined as the difference between the numerical solution for the non-stochastic cell length $L(\theta )$ and the statistical length $L_s$:

22 $$\begin{aligned} J(\theta )=\left\| L(\theta )-L_s\right\| . \end{aligned}$$

With the PSO we search the parameter values $\theta$ that allow the generation of numerical solutions that are as close as possible to the target values. More specifically, the desired solutions are the constant statistical lengths for RECs and UECs and the square wave for TECs, as described in Sect. 3.1.

An important aspect to emphasize is that the search for parameter values was conducted in specific ranges in order to avoid unphysical values. Before performing the optimization, a systematic sensitivity analysis was carried out with the aim of finding the most appropriate intervals of values where the optimal parameters would be expected to lie. We also note that the optimization was performed on the non-stochastic version of the problem, while the numerical simulations were subsequently performed on the stochastic system. This was possible because of the zero mean property of the Brownian motion. 

##### TECs:

In the case of TECs, the PSO was applied with the aim of determining $M_1$, $M_2$, $\lambda$, and $c_{eq}$, while $M_3$ was fixed a priori at $10^{-5}$. This choice was made after having observed that the desired oscillations arise for sufficiently low values of $M_3$. With the use of the optimizer, the following values were obtained: $M_1^*=7.4\times 10^4$, $M_2^*=6.4$, $c_{eq}^*=2.26$, and $\lambda^* =8.75\times 10^{-4}$. Solving the system of equations using these optimized values produces realistic outcomes whereby the desired amplitude of oscillations, the average tumbling phases, and the lengths of the tumbling intervals are all accurately reproduced. As illustrated in Fig. 6, the drop in cell length is accompanied by reduction in F-actin and ATP concentration, while an increase in ATP levels is associated with cell elongation and F-actin augmentation. These results are consistent with the postulated coupling between intracellular ATP levels and changes in cell elongation. The optimized parameters with the corresponding units are listed in Table 3.

**Fig. 7:**
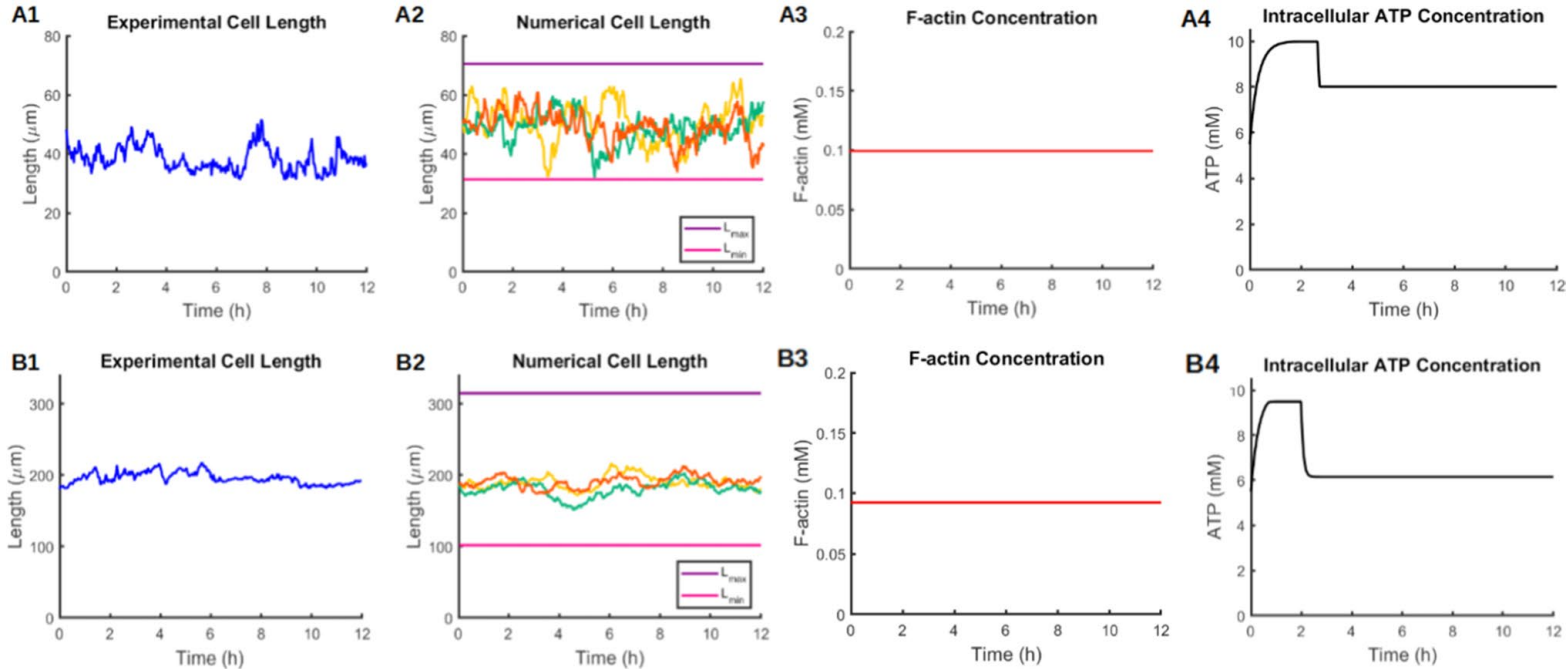
Top) RECs. Comparison between experimental cell length (A1) and Numerical cell length (A2), intracellular F-actin concentration (A3) and intracellular ATP concentration (A4) both numerically obtained. Bottom) UECs. Comparison between experimental cell length (B1) and Numerical cell length (B2), intracellular F-actin concentration (B3) and intracellular ATP concentration (B4) both numerically obtained. Computations performed over a time interval of duration $T=12$ h

The numerical results obtained with the calibration procedure described above do indeed reproduce the behavior of the “statistical” TECs in terms of maximum and minimum lengths, tumbling phase, and length of tumbling interval. It should be noted, however, that the experimental lengths exhibit a non-periodic behavior. In fact, experimental data show that cells can exhibit different tumbling phase durations ranging from as short as 10 min to as long as 4 hours. A possible approach to account for this non-periodic behavior in the current model is to “break” the hysteresis which is responsible for the periodicity of the solution. Efforts in this direction showed that non-periodic solutions were indeed attainable; however, they required the use of unrealistic parameter values and were consequently not used. It is anticipated, however, that with additional experimental data, a more refined formulation of the equations can allow us to obtain more realistic non-periodic results. In conclusion, the results obtained for TECs (Fig. 6) reproduce cell statistics and provide a first classification of the parameters; however, additional work is needed in order to provide a more complete description of the tumbling phenomenon.

##### RECs and UECs:

For RECs and UECs, the PSO was applied in order to find the best fit for $M_1, M_2, M_3$, and $c_{eq}$, while $\lambda$ was set to the same value as that obtained for TECs. The optimized values generated by the calibration procedure for both RECs and UECs are shown in Table 3. In both cases, the algorithm identified the best fit for the listed parameters in order to reproduce the statistical length, namely the average lengths shown in Fig. 5 (right) A2 and B2.

Once the parameter values were established, subsequent simulations were conducted on the stochastic version of the system. In light of the mean reverting property of the OU process (see Sect. 2.2.1), different oscillations of cell length around the statistical length can be obtained by varying the parameter $\phi$. A comparison between the experimental length and the computed lengths in the REC case is shown in Fig. 7 (A1 and A2). The different length profiles shown in Fig. 7 A2 were computed by solving the stochastic equation for cell length using randomly generated normal distributions that all meet the requirement of a zero mean and standard deviation equal to one. This allows the generation of a potentially infinite number of different length profiles.

**Table 3:** List of computed and optimized values for the three cell categories. Parameters with asterisk are the optimized ones

Symbol	TECs	RECs	UECs	Units	Meaning
$a_{\min }$	$5\times 10^{-2}$	$5\times 10^{-2}$	$5\times 10^{-2}$	mM	F-actin minimum concentration
$a_{sat}$	$2.5\times 10^{-2}$	$2.5\times 10^{-2}$	$2.5\times 10^{-2}$	mM	F-actin saturation concentration
$k_1$	$1.04\times 10^3$	$1.04\times 10^3$	$1.04\times 10^3$	$\text{s}^{-1}$	F-actin convergence rate to $a_m$
$k_2$	142.2	404	351.8	$\text{s}^{-1}$	F-actin convergence rate to $a_M$
$$\psi$$	$$5.6\times 10^3$$	$$2.4\times 10^3$$	752	$$\hbox {mM}^{-2}$$$$\text{s}^{-1}$$	Contractility constant
$M^*_1$	$7.4\times 10^4$	$2.15\times 10^2$	$2.15\times 10^2$	Dimensionless	Modulator of length-induced release
$M^*_2$	6.4	$1.6\times 10^{-2}$	$1.6\times 10^{-2}$	Dimensionless	Modulator of F-actin equilibria
$M^*_3$	$10^{-5}$	$8.5\times 10^{-4}$	$8.5\times 10^{-4}$	mM$^{-1}$	Hysteresis slope modulator
$\lambda ^*$	$8.75\times 10^{-4}$	$8.75\times 10^{-4}$	$8.75\times 10^{-4}$	${\text{s}^{-1}}$	ATP production rate
$c^*_{eq}$	2.26	8.07	8.07	mM	Equilibrium concentration for ATP

The results presented in Fig. 7 A2 were obtained by increasing the $\phi$ coefficient to 10. We recall that the optimization was performed on the non-stochastic version of the cell length equation, with the aim of reproducing a constant steady-state cell length (matching that obtained experimentally). To do so, we used the reference value $\phi =3\times 10^{-2}$. This constant steady-state length represents the average of the stochastic process. When the stochastic component is added, it is crucial to vary $\phi$ in order to obtain length profiles far from the average that resemble those in Fig. 7A1. These results indicate that the current model formulation is capable of capturing different cell length profiles that resemble those obtained experimentally and that depending on the value of $\phi$, different variances for cell length can be obtained.

Figure 7A3 and A4 depicts the computed time evolution of F-actin and intracellular ATP for RECs. The F-actin level is constant in time, consistent with the fact that the cell length remains constant. Intracellular ATP concentration, on the other hand, exhibits an initial transient phase before attaining a constant steady state value. Figure 7 (B1–B4) depicts the model results for the case of UECs. Panel B2 illustrates numerically computed cell length profiles for three runs of the algorithm and for $\phi =10$ in a manner similar to that used for RECs. Comparison with the experimental results (panel B1) shows that the model is capable of closely reproducing the experimental behavior.

The optimized parameter values for RECs and UECs are listed in Table 3. For both RECs and UECs, the initial conditions for F-actin were chosen to be equal to the F-actin values associated with the corresponding $k_1$ and $k_2$. The initial cell length was set to the statistical length, while an initial mean ATP concentration was imposed. In the case of TECs, the initial condition for F-actin was taken as the minimum concentration, while the initial condition for cell length was taken as $L_{\min }$. The initial condition for intracellular ATP was taken as an intermediate value. An important observation in Fig. 7 is that the same F-actin and intracellular ATP profiles can lead to different cell length profiles. This can be explained by the fact that for a certain range of model parameter values, the equation governing cell length (Eq. 8) becomes decoupled from the F-actin and ATP equations (Eqs. 1 and 14).

**Fig. 8:**
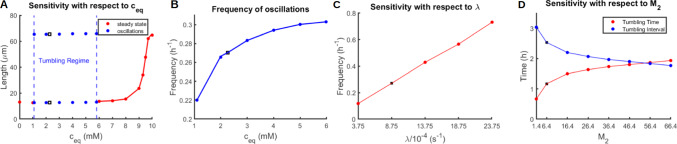
Sensitivity analysis for TECS. (**A**) In red the steady states assumed by cell length when increasing and decreasing $c_{eq}$, in blue the up an down points delineate the amplitude of oscillations in the interval [1.1, 5.98]. (**B**) The frequency of the oscillations emerging when $c_{eq}\in [1.1, 5.98]$. Sensitivity of parameter $\lambda$ (**C**) and $M_2$ (**D**). The black squares indicate the optimized values

**Fig. 9:**
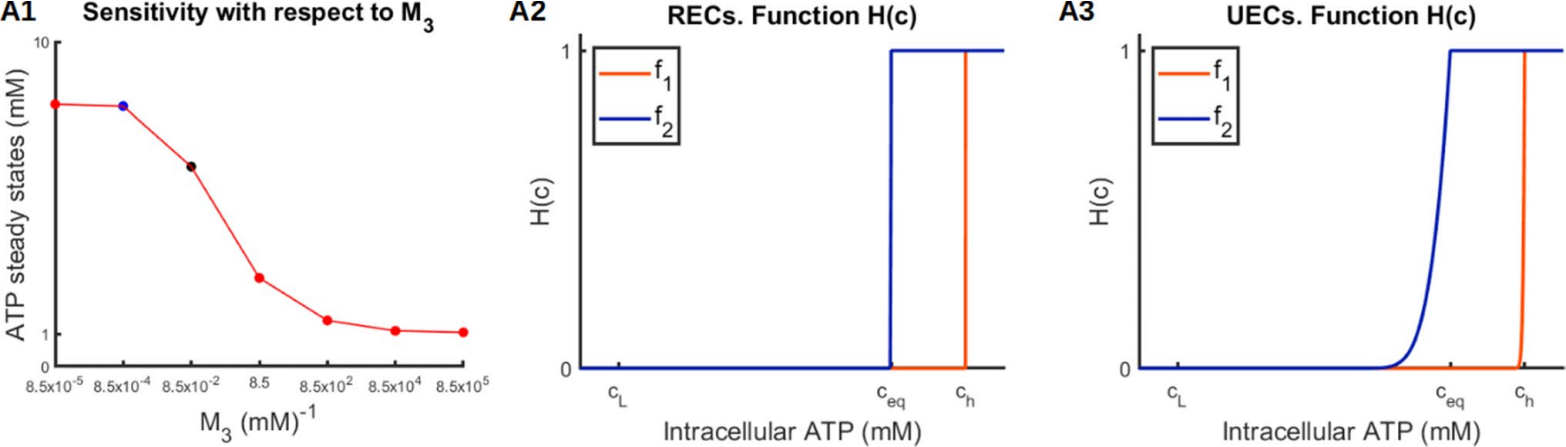
A1) Sensitivity analysis of the parameter $M_3$. Increasing values of $M_3$ correspond to decreasing values for the ATP steady state. The blue point indicates the value assumed in correspondence of the optimal parameter for RECs, while the black point the one for the optimal parameter for UECs. Hysteresis functions related to RECs (A2) and to UECs (A3)

**Fig. 10:**
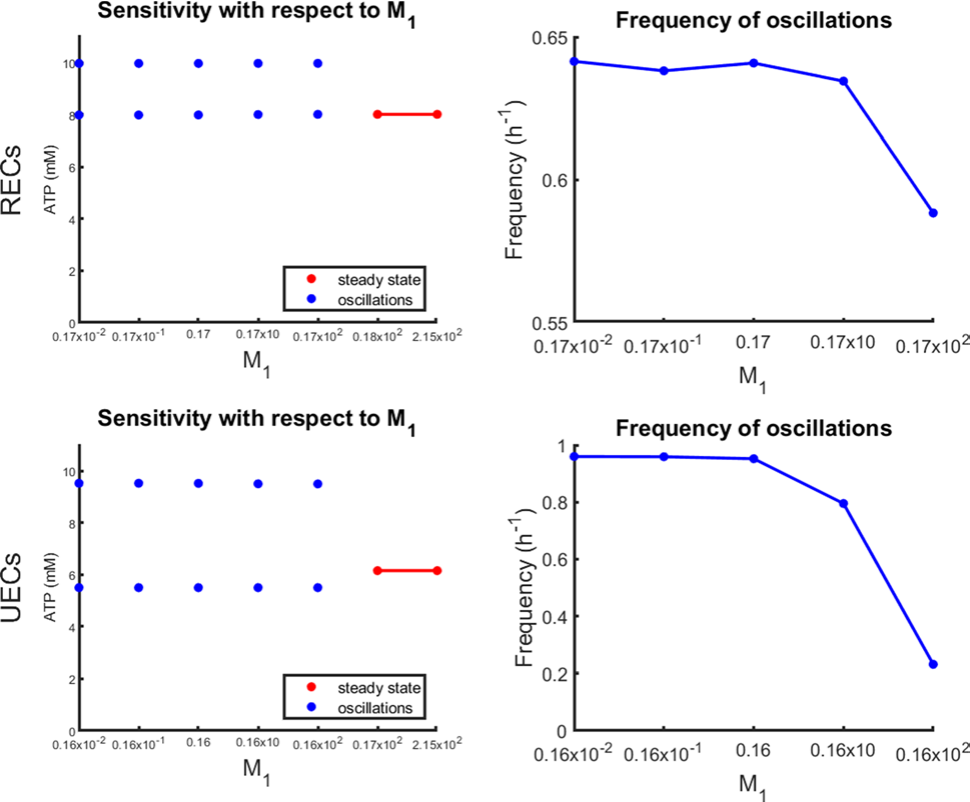
Sensitivity plots for the parameter $M_1$. RECs (Top), UECs (Bottom)

#### Sensitivity analysis

We have performed a detailed sensitivity analysis, in order to establish which parameters have the most influence on the model predictions. In the interest of brevity, the results of this analysis are only partially shown. Of particular interest in the sensitivity study is the determination of the parameters that govern a potential transition from one cell length phenotype to another. The approach used in the sensitivity analysis was to vary one parameter at a time over a large range while maintaining all the other parameters constant at their baseline values given in Table 3.

The results of the sensitivity analysis revealed that the transition between TECs, where cell length changes periodically in time, and the other two phenotypes (RECs and UECs), where cell length remains constant, is governed principally by the three model parameters $c_{eq}$, $M_2$, and $M_3$. More specifically, TECs are only observed for intermediate values of $c_{eq}$ (approximately 1 to 6 mM) combined with sufficiently high values of $M_2$ ($>0.1$) and relatively low values of $M_3$ ($<10^{-4}$). Furthermore, when tumbling occurs, its detailed characteristics, namely the tumbling frequency and the duration of each tumbling episode, are driven primarily by the parameters $c_{eq}$, $\lambda$, and $M_2$.

Figure 8 summarizes some of these findings. Panel A depicts the dependence of cell length on $c_{eq}$ and demonstrates that $c_{eq}$ is indeed a bifurcation parameter that allows transitioning between the tumbling and non-tumbling (constant length) regimes. Within the tumbling regime, cell length oscillates between 13 and 65-μm, whereas outside this regime, cell length increases rapidly with $c_{eq}$. Panels B and C show that for the tumbling regime, the tumbling frequency increases with both $c_{eq}$ and $\lambda$. Finally, panel D demonstrates that the average time per tumbling cycle increases with $M_2$, whereas the average time interval between consecutive tumbles correspondingly decreases.

While $c_{eq}$ and $M_2$ determine critical aspects of the tumbling behavior as shown above, the sensitivity analysis revealed that $M_1$ and $M_3$ have their principal impact on intracellular ATP levels without necessarily affecting cell length. More specifically, increasing $M_3$ (while holding all other parameter values constant) leads to lower steady-state intracellular ATP levels (Fig. 9), whereas decreasing $M_1$ induces intracellular ATP oscillations whose frequency decreases with $M_1$ (Fig. 10). Nevertheless, these oscillations do not produce real tumbling phases.

**Fig. 11:**
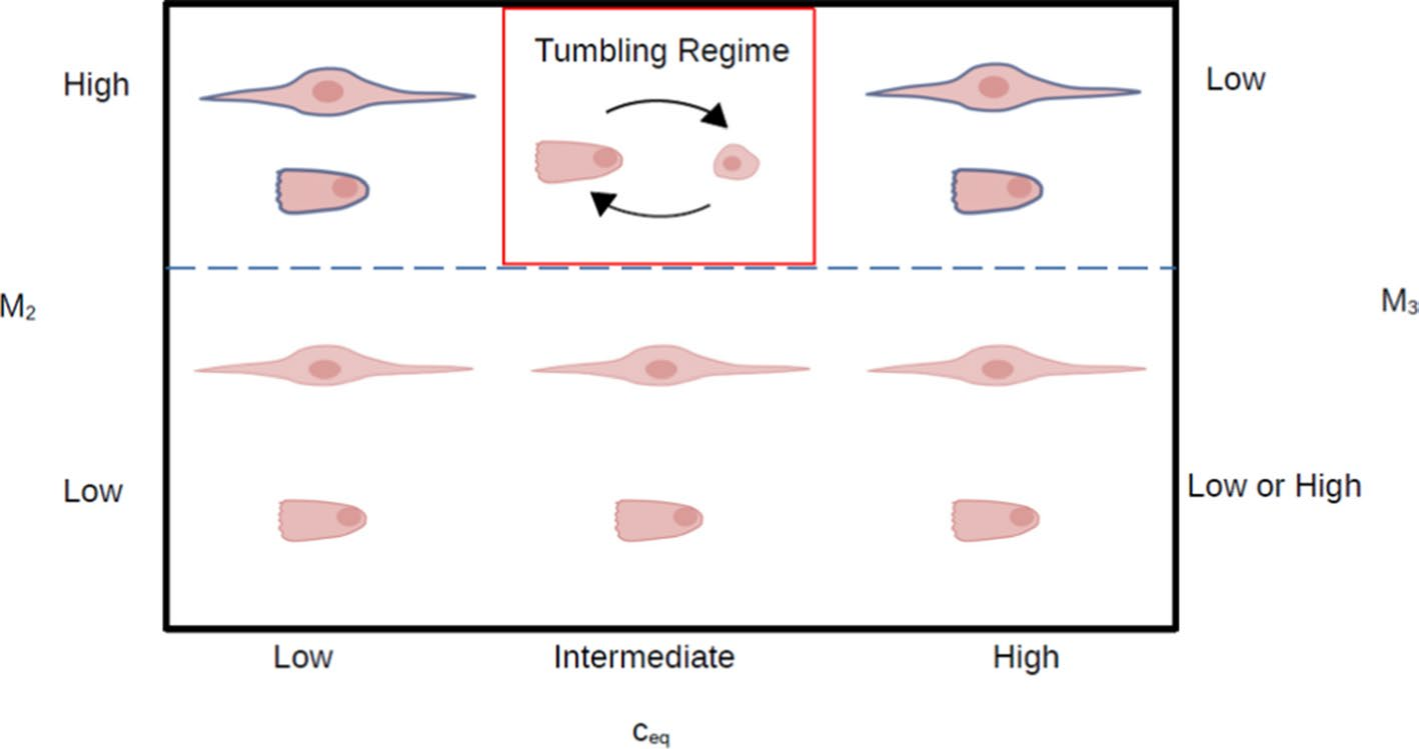
Different cell states according to parameters variations. The cells with the blue contour indicate that, their lengths before reaching the steady states, experience a transient phase

Figure 11 provides a schematic “phase diagram” that identifies where in the parameter space the tumbling behavior is predicted to occur. As already indicated, tumbling requires intermediate values of $c_{eq}$ in combination with sufficiently large values of $M_2$ and sufficiently low values of $M_3$. It is important to notice, that considering low and high values of $c_{eq}$, RECs and UECs appear, but before reaching the constant steady state, they experience a transient phase. For this reason in Fig. 11, the countours of these cells are in blue.

## General remarks

Table 3 shows the results obtained using the PSO algorithm. We can observe that $M_1$, $M_2$ and $M_3$ are the same for RECs and UECs, but different for TECs. According to the value of $M_2$, the *K* function can change its slope, modifying its concavity and as a consequence, the dependence between F-actin and cell length. Specifically, small values of $M_2$ produce a *K* curve that tends to the maximum equilibrium regardless of ATP variations, while greater values of $M_2$ allow a switch from one equilibrium to the other, permitting generation of oscillations. This is the reason why TECs have a different $M_2$ value. Parameter $M_3$ is related to the slope of the *H* function and its role needs to be contextualized in relation with $M_2$.

- If $M_2$ is low, the constant state is achieved, because the equilibrium is immediately reached and different values of $M_3$ might reflect different equilibria for the ATP solution. This behavior is the consequence on the one hand of the decoupling of the first two equations from the ATP equation (due to low $M_2$) and on the other hand on the shape of the hysteresis function. Higher values of $M_3$, producing smooth hysteresis curves, allow ATP loss that lowers the ATP equilibria. In this case, we have made a distinction between RECs and UECs, assuming that lower ATP levels correspond to UECs, while the higher levels correspond to RECs.
- In the hypothesis that $M_2$ is high, we might have two different situations: if $M_3$ is low, oscillations emerge, while if $M_3$ is high, ATP becomes constant. This last case is excluded from our discussion because F-actin, cell length and ATP reach an equilibrium value that corresponds to their absolute minimum and therefore this case is meaningless from the experimental point of view.

In conclusion, the parameters $M_2$ and $M_3$ have emerged as two principal discriminating parameters in cell behavior.

Another important parameter is $c_{eq}$, which appears in the hysteresis function. We have observed that changes in $c_{eq}$ reflect in changes in the ATP steady state due to its effect on the *H* function. However, we have preferred to focus on the discrimination based on $M_3$, because our hypothesis is based on the idea that RECs maintain high ATP levels, without releasing much ATP, compared to UECs for which the ATP release is more consistent although the ATP levels are maintained in an intermediate range. This hypothesis is satisfied when changing $M_3$ and not $c_{eq}$. In addition,

- if the parameter $M_2$ is low, the constant state is achieved, and in this context the parameter $\lambda$ serves only to control how fast the equilibrium is reached.
- When $M_2$ is high, both equilibria are reached, the oscillations appear and their frequency is determined by $\lambda$: higher values of $\lambda$, correspond to an increase in oscillations. Besides, the parameter $M_2$ has the additional role of controlling the duration of the steady phases in the TEC case: larger values of $M_2$ correspond to a longer tumbling phase. A thorough analysis of the effects of $\lambda$ and $M_2$ is presented in Sect. 3.2.3. Two other important parameters are $c_{eq}$ and $M_3$, related to the shape of the hysteresis function.

Lastly, the parameter $M_1$ does not appear to play a predominant role in cell behavior. We have only observed the appearance of oscillations in ATP (not in F-actin and cell length) for RECs and UECs for some values of $M_1$. The parameters related to the ATP equation were found through optimization. Analysis of these results is described in Sect. 3.2.3.

## Discussion

The mathematical model described in the present work was intended to provide a possible explanation for the experimental observation that ECs cultured on 15-μm-wide line patterns exhibit different migration phenotypes, referred to here as RECs, UECs, and TECs, that are associated with different cell length dynamics. The basic idea underpinning the model is that EC length is driven primarily by the coupled effects of intracellular ATP levels and F-actin organization. More specifically, we wanted to test the hypothesis that RECs and UECs were associated with fairly stable levels of intracellular ATP and F-actin levels, whereas the TEC phenotype was associated with oscillations in intracellular ATP that drive the periodic changes in EC shape observed experimentally.

The line patterns used here are intended to provide an idealized mimic of anisotropic contact guidance cues that direct cellular migration. In blood vessels *in vivo*, such cues would exist due to the anisotropic organization of the fibers of the extracellular matrix. In the context of vascular grafts, line patterns may represent anisotropies in the structural organization of these grafts. Because complete and rapid endothelial coverage of vascular grafts is essential for the success of grafting procedures, understanding EC migration on line patterns promises to provide insight into connections between graft surface architecture and the performance of these grafts in terms of the efficiency of endothelialization.

The model that was developed takes the form of a coupled set of differential equations that describe the dynamics of intracellular ATP, F-actin, and cell length. Stochasticity is incorporated into the equation governing the dynamics of cell length in order to capture experimentally observed small-scale and high-frequency fluctuations in cell length. In this framework, it is also important to notice the non-periodic nature of the tumbling interval in the case of TECs. However, the variations in tumbling interval length for any one cell are significantly larger than the small-scale variations in cell length captured by the stochastic term added to the governing equations. While we were able to estimate some of the model parameters either from our experiments or from the literature, a number of parameters remained unknown. To obtain best-estimate values for these parameters, we used the experimental results to establish the length dynamics of a “characteristic” EC for each of the three phenotypes, and we implemented an optimization scheme that provided best-fit values for these parameters. The results demonstrate that the proposed model is capable of generating profiles of cell length dynamics that closely match those observed experimentally. Different ranges of model parameters lead to behavior that resembles that of the three observed EC phenotypes. Furthermore, a detailed sensitivity analysis revealed which model parameters dictate which features of the observed dynamics for each of the different phenotypes.

Although the present findings suggest that the hypothesis that the different EC phenotypes observed on line patterns are driven by different profiles of intracellular ATP and F-actin is plausible, they do not provide definitive evidence for this hypothesis. All that can be said at this point is that the results are consistent with this hypothesis. Validating the hypothesis awaits experimental measurements of the dynamics of intracellular ATP levels in live ECs. Making such measurements is not a simple task. A recent study suggests that a Foerster resonance energy transfer-(FRET-) based biosensor may provide sufficient sensitivity and dynamic range for such measurements (Morciano et al. 2019); however, it remains to be determined if this technique can be used for ECs cultured on line patterns. Naturally﻿, there are other possible explanations for the occurrence of the tumbling-like behavior of ECs on line patterns. One particularly intriguing possibility is that the tumbling phase where the cells round up followed by spreading, polarization, and directed migration might represent a form of “frustrated division”. Cells typically round up prior to dividing (Ramkumar and Baum 2016), and the two daughter cells subsequently spread and polarize. It would be interesting to explore if the physical confinement conferred upon the cells by the line pattern prevents the process of cell division from completion. This hypothesis certainly merits future investigation.

The experimental results reported in the paper were for a 15-μm-wide fibronectin-coated line pattern. To assess how general this result is, we have conducted limited experiments on additional line widths, namely 5, 10, and 30-μm. Preliminary results suggest that reducing the line width does not significantly change the frequency of the TEC phenotype ($18\%$ TECs on 5-μm-wide lines vs. $21\%$ on 15-μm-wide lines). However, increasing line width to 30-μm appears to significantly reduce the incidence of tumbling ($3\%$ TECs). We have also conducted limited experiments on Type I collagen-coated line patterns (15-μm-wide) and observed a moderate increase in the incidence of tumbling relative to 15-μm-wide fibronectin-coated lines ($32\%$ vs. $21\%$ during 12-hr recordings). These findings need to be confirmed in additional future experiments.

## Conclusions

We have developed a model that describes the evolution of cell length with time for EC’s cultured on narrow line patterns that confer physical confinement onto the cells. The model involves a system of three coupled stochastic differential equations that represent the time evolution of F-actin, cell length, and intracellular ATP concentration. The model is shown to be able to capture the different types of behavior observed experimentally, namely runner, undecided and tumbling-like ECs.

A limitation of the current model is the absence of experimental data for F-actin or intracellular ATP concentration. While some parameters were obtained from literature, others were obtained from an optimization algorithm that provided the best fit with experimental data on the evolution of length in time. Thus, the current model is able to provide a classification of the three behaviors based on different parameter choices. Although a partial categorization of the cell phenotypes is possible, the biological mechanisms behind their occurrence remain unknown. Future work will focus on providing experimental evidence for the involvement of ATP as well as on extending the modeling to include cellular polarization and migration.

## Appendix

### Parameter estimation from experiments and modeling

Table 4 recapitulates the information provided in Tables 1, 2 and 3 in the main text. The first parameters that need to be estimated are the rate constants $k_1$ and $k_2$, which appear in the *K*(*c*) function of Eq. (1). These values are significant players in determining the equilibria of the F-actin equation and thus also the equilibria of the length equation. To determine values for these rate constants, the modeling can be used subject to constraints imposed by known data. According to Alberts et al. (2002), the intracellular F-actin concentration lies in the range of 0.05 mM to 0.2 mM. Thus, we impose that the F-actin concentration needs to be greater than 0.05 mM at all times. Consequently, using Eq. (3), it follows that:

$$\begin{aligned} a_{\min }=\dfrac{ka_h}{k+k_1}\ge 0.05\: \hbox{mM}, \end{aligned}$$

and

23 $$\begin{aligned} k_1\le \biggl (\dfrac{a_h-0.05\: \hbox{mM}}{0.05\: \hbox{mM}}\biggr )k. \end{aligned}$$

Substituting $a_h=0.2$ mM, it follows that $k_1\le 3k$, where *k* is the estimated rate of F-actin production (Pollard,Mogilner and Oester 1986). For the numerical simulations, $k_1$ was chosen to be equal to $3k = 1.04\times 10^3$ ${\text {s}^{-1}}$, corresponding to $a_{\min }=0.05$ mM. It is important to note that the choice of $k_1$ is not single-valued since the relation (23) applies for all values smaller than 3*k*. Nonetheless, convenient assumptions had to be made in order to obtain consistent results.

**Table 4:** List of parameter values for each cell category

Symbol	TECs	RECs	UECs	Units	Meaning
$a_{h}$	$2\times 10^{-1}$	$2\times 10^{-1}$	$2\times 10^{-1}$	mM	F-actin homeostatic value
$a_{\min }$	$5\times 10^{-2}$	$5\times 10^{-2}$	$5\times 10^{-2}$	mM	F-actin minimum concentration
$a_{sat}$	$2.5\times 10^{-2}$	$2.5\times 10^{-2}$	$2.5\times 10^{-2}$	mM	F-actin saturation concentration
*k*	347	347	347	$\text{s}^{-1}$	F-actin Polymerization Rate
$c_{h}$	10	10	10	mM	ATP homeostatic value
$c_{L}$	1	1	1	mM	ATP minimum concentration
$k_1$	$1.04\times 10^3$	$1.04\times 10^3$	$1.04\times 10^3$	$\text{s}^{-1}$	F-actin convergence rate to $a_m$
$v_p$	25	25	25	${\upmu } \text{m} \text{s}^{-1}$	Protrusion Velocity
$\lambda$	$8.75\times 10^{-4}$	$8.75\times 10^{-4}$	$8.75\times 10^{-4}$	$\text{s}^{-1}$	ATP production Rate
$k_2$	142.2	404	351.8	$\text{s}^{-1}$	F-actin convergence rate to $a_M$
$c_{eq}$	2.26	8.07	8.07	mM	ATP equilibrium concentration
$\phi$	$3\times 10^{-2}$	10	10	Dimensionless	Friction coefficient
$M_1$	$7.4\times 10^4$	$2.15\times 10^2$	$2.15\times 10^2$	Dimensionless	Modulator of length-induced release
$M_2$	6.4	$1.6\times 10^{-2}$	$1.6\times 10^{-2}$	Dimensionless	Modulator of F-actin equilibria
$M_3$	$10^{-5}$	$8.5\times 10^{-4}$	$8.5\times 10^{-4}$	$\hbox {mM}^{-1}$	Hysteresis slope modulator
$\psi$	$5.6\times 10^3$	$2.4\times 10^3$	752	$\hbox {mM}^{-2}\text{s}^{-1}$	Mathematical expedient/No physical meaning

With regards to the parameter $k_2$, the relation for the maximum F-actin (see 4) could not be used because it leads to a trivial result (we would find $k_2\ge 0$). Thus, other constraints needed to be considered. In Sect. 2.2.1, we have seen that length equilibria are provided through the relation:

24 $$\begin{aligned} L=\dfrac{v_p}{\sigma (a)}=\dfrac{v_p}{\psi a^2 e^{-a/a_{sat}}}, \end{aligned}$$

which applies for both the stochastic and the non-stochastic cases (because the process we are studying has a zero mean). From the definition of $\sigma (a)$ and from the previous choice of $k_1$, it follows that $a_{sat}=a_{\min }/2=0.025$ mM. In addition, the minimum length for each cell phenotype which was estimated from the experiments (Table 2) can be used in the following manner:

25 $$\begin{aligned} L_{\min }=\dfrac{v_p}{\psi a_{\min }^2 e^{-2}}, \end{aligned}$$

and $\psi =\dfrac{v_p}{L_{\min } a_{\min }^2 e^{-2}}$ is derived. An important observation is that $\psi$ can change either due to changes in the value of $a_{\min }$, but in the current work $a_{\min }$ was maintained constant at 0.05 mM, or due to changes in the values of $L_{\min }$ which depend on cell phenotype (24). Substituting $\psi$ into relation (24), it follows that:

26 $$\begin{aligned} L=L_{\min }\biggl (\dfrac{a_{\min }}{a}\biggr )^2e^{-2+a/a_{sat}}, \end{aligned}$$

and this relation can be written as:

27 $$\begin{aligned} a^2=\biggl (\dfrac{L_{\min }}{L}\biggr )a_{\min }^2e^{-2+a/a_{sat}}. \end{aligned}$$

The previous relation is nonlinear and the roots can be easily computed for each of the three cell phenotypes. In particular, since the parameter $k_2$ is related to the maximum attainable equilibrium for F-actin, when solving Eq. (27), *L* must be imposed to be equal to the maximum equilibrium that needs to be reached by cells. Specifically, for TECs *L* is imposed as the $L_{\max }$ derived from the experiments, while for RECs and UECs, since the lengths that need to be reproduced do not coincide with the maximum value of the range but rather with the statistical length, *L* will be imposed to be equal to this latter quantity.
